# Chronic cannabis use alters the spontaneous and oscillatory gamma dynamics serving cognitive control

**DOI:** 10.1002/hbm.26787

**Published:** 2024-07-18

**Authors:** Mikki Schantell, Jason A. John, Anna T. Coutant, Hannah J. Okelberry, Lucy K. Horne, Ryan Glesinger, Seth D. Springer, Amirsalar Mansouri, Pamela E. May‐Weeks, Tony W. Wilson

**Affiliations:** ^1^ Institute for Human Neuroscience Boys Town National Research Hospital Boys Town Nebraska USA; ^2^ College of Medicine University of Nebraska Medical Center (UNMC) Omaha Nebraska USA; ^3^ Department of Neurological Sciences UNMC Omaha Nebraska USA; ^4^ Department of Pharmacology and Neuroscience Creighton University Omaha Nebraska USA

**Keywords:** cannabis use disorder (CUD), CBD, magnetoencephalography (MEG), marijuana, phase locking, PLV

## Abstract

Regular cannabis use is associated with cortex‐wide changes in spontaneous and oscillatory activity, although the functional significance of such changes remains unclear. We hypothesized that regular cannabis use would suppress spontaneous gamma activity in regions serving cognitive control and scale with task performance. Participants (34 cannabis users, 33 nonusers) underwent an interview regarding their substance use history and completed the Eriksen flanker task during magnetoencephalography (MEG). MEG data were imaged in the time‐frequency domain and virtual sensors were extracted from the peak voxels of the grand‐averaged oscillatory interference maps to quantify spontaneous gamma activity during the pre‐stimulus baseline period. We then assessed group‐level differences in spontaneous and oscillatory gamma activity, and their relationship with task performance and cannabis use metrics. Both groups exhibited a significant behavioral flanker interference effect, with slower responses during incongruent relative to congruent trials. Mixed‐model ANOVAs indicated significant gamma‐frequency neural interference effects in the left frontal eye fields (FEF) and left temporoparietal junction (TPJ). Further, a group‐by‐condition interaction was detected in the left FEF, with nonusers exhibiting stronger gamma oscillations during incongruent relative to congruent trials and cannabis users showing no difference. In addition, spontaneous gamma activity was sharply suppressed in cannabis users relative to nonusers in the left FEF and TPJ. Finally, spontaneous gamma activity in the left FEF and TPJ was associated with task performance across all participants, and greater cannabis use was associated with weaker spontaneous gamma activity in the left TPJ of the cannabis users. Regular cannabis use was associated with weaker spontaneous gamma in the TPJ and FEF. Further, the degree of use may be proportionally related to the degree of suppression in spontaneous activity in the left TPJ.

## Graphical abstract

Regular cannabis use has sharply increased over the past decade due to widespread legalization, but the impact on the brain is poorly understood. Herein, we investigated whether such cannabis use alters neurophysiological activity using magnetoencephalography and found differences in spontaneous and oscillatory gamma activity in users that scaled with behavior.

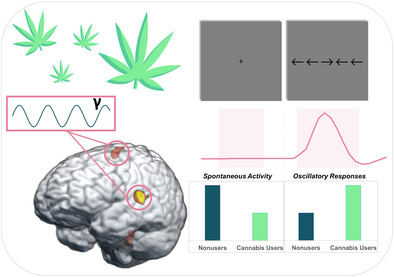

## 1 INTRODUCTION

Cannabis use has been associated with acute alterations in attention and executive function, though the long‐term effects of chronic cannabis use on cognition are less understood (Anderson et al., 2010; Broyd et al., 2016; Hunault et al., 2009; Lane et al., 2005; Ramaekers et al., 2006; Vadhan et al., 2007). The main psychoactive component in cannabis is Δ^9^‐tetrahydrocannabinol (Bloomfield, Hindocha, Green, Wall, Lees, & Petrilli, 2019; Chou et al., 2022), which is an agonist of the endocannabinoid CB_1_ and CB_2_ receptors (Howlett & Abood, 2017). Activation of CB_1_ receptors, which are highly concentrated in frontal and limbic regions in the cortex (Bloomfield, Hindocha, Green, Wall, Lees, Petrilli, Costello, et al., 2019), are more prevalent on GABAergic than glutamatergic neurons, and thereby are strategically positioned to modulate gamma activity (Katona et al., 1999; Puighermanal et al., 2009). Essentially, extensive evidence from cellular studies has demonstrated that gamma oscillations are generated by local networks of fast‐spiking parvalbumin‐expressing GABAergic interneurons (Bartos et al., 2007; Buzsáki & Draguhn, 2004; Buzsáki & Wang, 2012; Fries, 2009; Fries, 2015; Fries et al., 2007; Kim & Paredes, 2021; Salkoff et al., 2015; Singer, 1999; Uhlhaas et al., 2009; Uhlhaas & Singer, 2012; Vinck et al., 2013). This work has been further supported by neurophysiological studies in humans, which have linked local GABA concentrations measured using magnetic resonance spectroscopy to the peak gamma frequency in the same cortical regions (Edden et al., 2009; Gaetz et al., 2011; Kujala et al., 2015; Muthukumaraswamy et al., 2009). Specifically, these studies suggest that higher local GABA levels are associated with elevated peak gamma frequency (Edden et al., 2009; Gaetz et al., 2011; Muthukumaraswamy et al., 2009), though it remains unclear how GABA concentrations are related to cortical gamma power.

The endocannabinoid system has been shown to mediate neural activity by modulating CB_1_ receptors on GABAergic interneurons distributed across the cortex (Hajós et al., 2008; Hájos et al., 2000; Katona et al., 1999; Morgan et al., 2008; Robbe et al., 2006; Skosnik et al., 2012). Thus, exogenous CB_1_ receptor agonists such as THC are thought to disrupt gamma dynamics, which are largely governed by GABAergic interneurons, and this is associated with downstream implications including a greater suppression in spontaneous power in both acute and abstinent regular cannabis users (Böcker et al., 2010; Herning et al., 2003; Ilan et al., 2004; Skosnik et al., 2012; Skosnik et al., 2016). Given these potential ramifications, linking chronic cannabis use to alterations in gamma activity is of critical interest, as alterations in GABAergic signaling can lead to large‐scale changes in neural population dynamics, including spontaneous neural activity (e.g., seemingly random discharges, resting‐state neuronal rhythms, and fluctuations in dendritic currents in the absence of explicit exogenous inputs) during the pre‐stimulus baseline period (referred to as “spontaneous activity” from here on out) and oscillatory neural activity (i.e., spectrally‐specific stimulus‐related changes in activity that are time‐locked to the onset of a stimulus) during task processing (Skosnik & Cortes‐Briones, 2016).

Cognitive control is a component of executive function that involves engaging in goal‐oriented behaviors to set and maintain task‐relevant goals to solve complex or novel tasks, such as overcoming prepotent responses and suppressing task‐irrelevant representations (Miller & Cohen, 2001). The cognitive control construct is comprised of several subconstructs including goal selection, updating, representation and maintenance, response selection, inhibition and selection, and performance monitoring (Botvinick et al., 2001). These operations employ the network‐level coordination of neural oscillatory activity distributed across the frontoparietal, cingulo‐opercular, and salience networks in the brain, which work together to support processes related to resolving conflict and detecting salient stimuli (Dosenbach et al., 2006; Halassa & Kastner, 2017). A common task used to assess cognitive control is the Eriksen flanker task (Eriksen & Eriksen, 1974), which requires participants to focus on a target stimulus and ignore the surrounding non‐target (i.e., “flanker”) stimuli that are either congruent to the target stimulus (e.g., arrows pointing in the same direction) or incongruent (e.g., arrows pointing in the opposite direction). For example, in the arrow‐based version of the flanker task, participants are instructed to respond whether the middle arrow is pointing to the left or to the right, thus requiring a greater suppression of task‐irrelevant information during the incongruent relative to the congruent condition. Studies using magnetoencephalography (MEG)/EEG have shown stronger frontal theta and parieto‐occipital alpha oscillations during incongruent relative to congruent trials (Cavanagh et al., 2009; Cavanagh & Frank, 2014; Cohen & Cavanagh, 2011; Cohen & van Gaal, 2014; Gulbinaite et al., 2014; McDermott et al., 2017; Nigbur et al., 2011; Nigbur et al., 2012; Padrão et al., 2015; Pastötter et al., 2013), but surprisingly, few studies have looked at spontaneous and oscillatory gamma activity serving cognitive control during the flanker task (Wiesman et al., 2020) since gamma oscillations in the PFC have been repeatedly associated with attention, cognitive control, and other higher order processes (Baldauf & Desimone, 2014; Doesburg et al., 2008; Marshall et al., 2015).

Studies looking at the impact of cannabis use on neural activity serving cognitive control processes have identified weaker activation in frontoparietal and cerebellar regions among regular cannabis users relative to nonusers (Abdullaev et al., 2010; Chang et al., 2006). Additionally, MEG studies of cognitive control and attention have found that regular cannabis users have aberrant theta responses in the right occipital cortex, weaker spontaneous and stronger oscillatory theta in the inferior frontal gyri and altered occipito‐frontal functional connectivity (Rangel‐Pacheco et al., 2021; Springer et al., 2023). These studies also showed that cannabis users maintained performance levels that did not statistically differ from nonusers, which may indicate that cannabis users employ compensatory neural processing during higher‐order cognitive tasks (Rangel‐Pacheco et al., 2021; Springer et al., 2023). However, in this context, the gamma oscillatory dynamics have not been widely examined, and thus, whether they are perturbed with regular cannabis use remains unknown. One previous study focusing on gamma activity in chronic cannabis users showed that cannabis users have suppressed spontaneous gamma activity in the somatosensory cortex and impaired oscillatory gating of somatosensory information within the same cortical tissue relative to nonusers, suggesting that chronic cannabis use is indeed associated with aberrations in gamma activity and cortical inhibitory processing (Arif, Wiesman, et al., 2021). Overall, these findings suggest that chronic cannabis use is associated with changes in various aspects of cognitive control and may be related to the dysregulation of GABAergic signaling and altered functional connectivity, thus underscoring the importance of investigating cannabis‐related alterations in the gamma dynamics serving higher‐order cognitive processes.

Although previous studies have found network‐level theta differences supporting cognitive control using the flanker task in cannabis users (Rangel‐Pacheco et al., 2021), no studies to date have examined whether regular cannabis use modulates the gamma dynamics serving cognitive control. Herein, we quantified the impact of regular cannabis use on the gamma‐specific oscillatory dynamics serving cognitive control using the flanker (Eriksen & Eriksen, 1974) paradigm in a large sample of regular cannabis users and nonusers. We hypothesized that conditional differences (i.e., the flanker interference effect) in gamma activity would be strongest in the frontoparietal cortices, and that excitatory/inhibitory imbalances would be reflected through sharply reduced spontaneous gamma activity in these regions in cannabis users relative to nonusers, which would scale with cannabis use metrics.

## 2 METHODS

### 2.1 Participants

A sample of 67 participants (33 nonusers, 34 cannabis users) who successfully completed a battery of neuropsychological assessments, structured substance use interview, and the flanker task during MEG were recruited from the Omaha metropolitan area. Participants in the cannabis group had used cannabis at least two to three times per week for the past six months and were using substances other than cannabis less than monthly. Nonusers were eligible for inclusion if they reported they had not used cannabis or any other illicit substances (other than alcohol) within the past year, and no history of frequent cannabis use (i.e., more than monthly use) in their lifetime. Full demographics by group are listed in Table 1. Exclusion criteria included any neurological or psychiatric disorder, history of head trauma, current pregnancy, or ferrous metallic implants that could interfere with MEG data acquisition. Further, participants using psychiatric, neurological, or other prescription medications known to strongly affect neural functioning (e.g., anticonvulsants, antipsychotics, immunosuppressives) were excluded from further participation in the study. The Institutional Review Board reviewed and approved this protocol. All participants gave written informed consent following detailed description of the study. The analysis for this study was not pre‐registered, and thus, the results of this study should be considered exploratory.

**TABLE 1:** Participant demographics and clinical indices.

	Nonusers *n* = 33	Cannabis users *n* = 34	*p*‐value
Age (years)	36.18 (11.21)	34.72 (10.33)	.586
Sex (male/female)	16/17	20/14	.396
AUDIT‐C score	2.52 (1.87)	3.64 (2.32)	.085
CUDIT‐R score	‐	13.24 (4.61)	‐
Learning T‐score	46.18 (11.40)	41.53 (10.58)	.088
Memory T‐score	49.85 (7.31)	45.94 (7.85)	.039
Executive function T‐score	52.18 (5.65)	47.42 (6.35)	.002
Attention T‐score	54.41 (7.52)	51.29 (8.26)	.112
Processing speed T‐score	53.42 (5.99)	51.00 (7.73)	.157
Motor dexterity T‐score	47.83 (8.30)	45.32 (7.18)	.190

*Note*: Means and standard deviations are displayed for all variables except sex, which is displayed as the number of males and females, respectively. Higher scores on the AUDIT‐C and CUDIT‐R are indicative of greater alcohol or cannabis use disorder symptomatology, respectively. AUDIT‐C: Alcohol Use Disorders Identification Test—Concise; CUDIT‐R: Cannabis Use Disorders Identification Test—Revised.

### 2.2 Neuropsychological assessment

All participants underwent a neuropsychological battery that assessed the following cognitive domains: *learning* (Hopkins Verbal Learning Test—Revised [HVLT‐R] Learning Trials 1–3) (Benedict et al., 1998), *memory* (HVLT‐R Delayed Recall and Recognition Discriminability Index) (Benedict et al., 1998), *executive functioning* (phonemic verbal fluency, semantic verbal fluency, Comalli Stroop Test Interference Trial, Trail Making Test Part B) (Comalli et al., 1962; Heaton et al., 2004), *processing speed* (Trail Making Test Part A, Wechsler Adult Intelligence Scale, Third Edition [WAIS‐III] Digit Symbol Coding, Comalli Stroop Test Color Trial) (Comalli et al., 1962; Heaton et al., 2004; Wechsler, 1997), *attention* (WAIS‐III Symbol Search, Comalli Stroop Test Word Trial) (Comalli et al., 1962; Wechsler, 1997), and *motor* (Grooved Pegboard—Dominant and Non‐Dominant Hands) (Heaton et al., 2004; Klove, 1953). Scores were corrected for demographic variables (e.g., age, education) using published normative data (Benedict et al., 1998; Comalli et al., 1962; Heaton et al., 2004; Wechsler, 1997) and were transformed to T‐scores. The T‐scores of tests within each cognitive domain were averaged together to create domain composite scores and are reported in Table 1.

### 2.3 Substance use assessments

All cannabis users completed substance use assessments including a thorough interview regarding their lifetime and current (within the last 12 months) substance use history using the NIDA Quick Screen (Version 1), NIDA‐Modified Alcohol, Smoking, and Substance Involvement Screening Test (NIDA‐modified ASSIST; Version 2), and Module E of the Structured Clinical Interview for the Diagnostic and Statistical Manual, 5th Edition (SCID‐5‐RV). Participants also completed self‐report questionnaires including the Cannabis Use Disorders Identification Test—Revised (CUDIT‐R), the Alcohol Use Disorders Identification Test—Concise (AUDIT‐C), and a customized cannabis use questionnaire detailing their cannabis use methods and history. Participants also provided a sample for urinalysis to confirm no recent use of other substances other than cannabis. Further, nonusers and cannabis users were interviewed regarding their past and current substance use using a standardized medical history interview, and alcohol use was assessed using the AUDIT‐C.

### 2.4 MEG experimental paradigm

**FIGURE 1:**
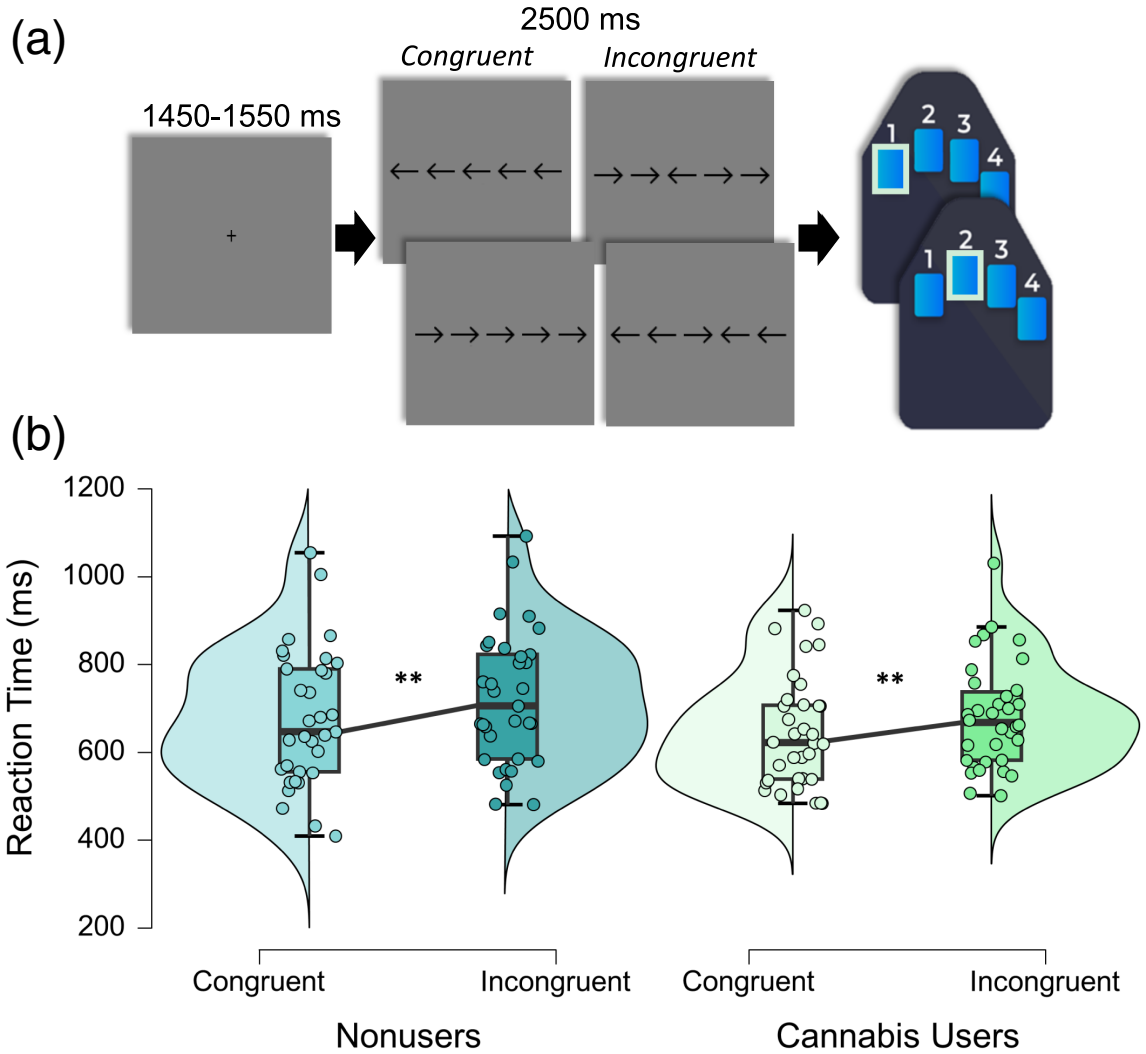
Flanker task paradigm and behavioral results. (a) An illustration of the Eriksen Flanker paradigm. Each trial had a fixation period lasting on average 1500 ms (variable ISI: 1450–1550 ms) and a stimulus‐presentation period lasting 2500 ms, which consisted of one of the four options displayed. (b) Behavioral results from the flanker task. Reaction time (RT; in ms) is displayed on the *y*‐axis by condition comparing nonusers (left) and cannabis users (right); both groups exhibited a significant condition effect (i.e., flanker effect), but there was not a significant main effect of cannabis group or a cannabis use‐by‐condition interaction. **p < .001.

Participants were seated in a nonmagnetic chair within a magnetically shielded room and completed a 14‐min arrow‐based flanker task with 200 pseudorandomized trials. A fixation cross was centrally presented for 1450–1550 ms, which was followed by the presentation of a row of five arrows for 2500 ms. Participants responded using their right hand whether the middle arrow was pointing to the left (index finger) or right (middle finger; Figure 1a). The trials were equally divided between congruent and incongruent conditions, with left and right arrows being equally represented in each of the conditions in a fixed pseudorandomized order. Reaction time and accuracy measures were collected and used for behavioral analysis.

### 2.5 MEG and MRI data acquisition

MEG recordings were conducted in a one‐layer magnetically shielded room with active shielding engaged. Neuromagnetic responses were acquired with an Elekta/MEGIN MEG system with 306 magnetic sensors (204 planar gradiometers, 102 magnetometers; Elekta, Helsinki, Finland) using a bandwidth of .1–.330 Hz, sampled continuously at 1 kHz. Each MEG dataset was individually corrected for head motion, and noise reduction was applied using the signal space separation method with a temporal extension (tSSS; correlation limit: .950; correlation window duration: 6 s; Taulu & Simola, 2006). Only data from the gradiometers were used for further analysis.

Structural T1‐weighted images were collected using a 3D‐fast‐field echo sequence on a Philips Achieva 3.0T X‐Series scanner with an eight‐channel head coil. MEG and MRI data processing closely followed previously reported pipelines (Wiesman et al., 2021; Wiesman & Wilson, 2020; Wilson et al., 2016). The structural MRI data were aligned parallel to the anterior and posterior commissures and transformed into standardized space. All MEG and MRI data were further processed in BESA (Research: V7.0; MRI: V2.0; Statistics: V3.0).

### 2.6 MEG coregistration and structural MRI processing

Prior to MEG recording, four coils were attached to the participant's head and localized with the three fiducial points and scalp surface using a 3‐D digitizer (Fastrak, Polhemus Navigator Sciences, Colchester, VT, USA). Once the participant was positioned for MEG recording, an electric current with a unique frequency label (e.g., 322 Hz) was fed to each coil, thus inducing a measurable magnetic field and thereby allowing each coil to be localized in reference to the MEG sensor array throughout the recording session. Since coil locations were also known in head coordinates, all MEG measurements could be transformed into a common coordinate system. With this coordinate system, each participant's MEG data were coregistered with their individual structural T1‐weighted MRI data prior to source space analyses using BESA MRI (Version 2.0; BESA). Following source analysis (i.e., beamforming), each participant's functional images were also transformed into standardized space using the transform that was previously applied to the structural MRI volume and spatially resampled.

### 2.7 MEG time‐frequency transformation

Cardiac and ocular artifacts were removed from the data using signal‐space projection (SSP), which was accounted for during source reconstruction (Uusitalo & Ilmoniemi, 1997). The resulting artifact‐corrected data were then band‐pass filtered from 0.5 to 150 Hz, notch filtered at 60 Hz, and divided into 2000 ms epochs (−500 to 1500 ms) with the baseline extending from −450 to −50 ms prior to the onset of the flanker stimulus (i.e., time 0.0 s). Epochs containing artifacts were rejected based on a fixed threshold method that was set per participant and supplemented with visual inspection. Briefly, in MEG, the raw signal amplitude is strongly affected by the distance between the brain and the MEG sensor array, as the magnetic field strength falls off sharply as the distance from the current source (i.e., brain) increases. To account for this source of variance across participants, as well as other sources of variance, we used an individualized threshold based on the signal distribution for both amplitude and gradient to reject artifacts. An average of 181.48 (SD = 12.56) segments were retained for further analyses, and there were no significant differences in the number of segments retained by condition (*t* = −.03, *p* = .976; congruent: mean = 90.75, SD = 6.70, incongruent: mean = 90.73, SD = 6.48) nor by cannabis use status (*t* = .33, *p* = .740; cannabis users: mean = 180.97, SD = 12.09; nonusers: mean = 182.00, SD = 13.20).

Artifact‐free epochs were transformed into the time‐frequency domain using complex demodulation (Kovach & Gander, 2016; Papp & Ktonas, 1977). Briefly, complex demodulation first transforms the signal into the frequency space, using a fast Fourier transform. This results in a frequency spectrum, inherently containing the same power and cross‐spectral information as the original signal. From here, this frequency spectrum is (de)modulated in a step‐wise manner to adopt the center frequency of a series of complex sinusoids with increasing carrier frequencies, in a process termed “heterodyning.” These resulting signals are then low‐pass filtered to reduce spectral leakage, and thus, the nature of this filter inherently determines the time and frequency resolution of the resulting data. For this study, the time‐frequency analysis was performed with a frequency‐step of 2 Hz and a time‐step of 25 ms between 30 and 100 Hz, using a 4 Hz low‐pass finite impulse response filter (Hoechstetter et al., 2004). The resulting spectral power estimations per sensor were averaged across trials to generate time‐frequency plots of mean spectral density. These sensor‐level data were then normalized with respect to the baseline power using a proportional approach (i.e., *[(Activity*_*active*_ *(t*,*f) − Activity*_*baseline*_ *(f))/Activity*_*baseline*_ *(f)] × 100%*).

### 2.8 Sensor‐level statistics

The resulting spectral power estimations per sensor were averaged across trials to generate time‐frequency plots of mean spectral density. These sensor‐level data were then normalized with respect to the mean baseline power (i.e., mean power during the −450 to −50 ms baseline period). Time‐frequency windows (2 Hz, 25 ms resolution) for subsequent source imaging were identified using a stringent two‐stage statistical analysis involving paired‐samples *t* tests against the power during the baseline period across all artifact free trials (i.e., congruent and incongruent), participants (i.e., cannabis users and nonusers), and the entire array of gradiometers from 30 to 100 Hz to focus on gamma‐frequency oscillations serving cognitive control. Pixels that were significant at the *p* < .005 level were then clustered with spectrally and temporally neighboring pixels that were also above the threshold and a cluster value was derived by summing the *t*‐values of all pixels in the cluster. To control for Type 1 error, these clusters were then subjected to nonparametric cluster‐based permutation testing to correct for multiple comparisons in stage two using 10,000 permutations per comparison and a corrected *p*‐value of .05 (Ernst, 2004; Maris & Oostenveld, 2007; Proskovec et al., 2018; Wiesman et al., 2018).

### 2.9 MEG source imaging

Time‐frequency resolved beamformer source images were computed using the dynamic imaging of coherent sources (regularization: singular value decomposition 0.0001%; Dalal et al., 2006; Gross et al., 2001; Van Veen et al., 1997) approach, which uses the cross‐spectral density data to calculate voxel‐wise estimates of neural power. Following convention, we computed noise‐normalized, source power per voxel (resolution: 4 × 4 × 4 mm) in each participant using active (i.e., task) and passive (i.e., baseline) periods of equal duration and bandwidth. The use of active and passive periods with comparable durations and bandwidths is essential, as it ensures that the dual‐state beamformer is not biased by the inclusion of different amounts of data in either window. Such images are typically referred to as pseudo‐t maps, with units (pseudo‐t) that reflect noise‐normalized power differences (i.e., active vs. passive) per voxel. This approach generated three‐dimensional participant‐level pseudo‐t maps per condition (i.e., congruent and incongruent), for each time‐frequency cluster identified in the sensor‐level analysis (i.e., gamma response from 62 to 70 Hz, 200 to 350 ms). The resulting beamforming maps were then transformed into standardized space (i.e., Talairach) and spatially resampled by applying the same transform that was applied to the native space structural images per participant.

### 2.10 Whole‐brain neural interference maps

To examine oscillatory activity specific to cognitive control, source images for the congruent condition were subtracted from the incongruent condition for each participant, resulting in whole‐brain maps of flanker interference activity per participant. These maps were averaged across all participants to generate a grand‐averaged map of the neural flanker interference effect. Peak voxel pseudo‐t values were then extracted from each participant's oscillatory map, separately by condition to evaluate group‐by‐condition interactions in oscillatory gamma power serving cognitive interference.

### 2.11 Peak voxel virtual sensor time series analyses

Virtual sensor data were computed by applying the sensor‐weighting matrix derived from the forward solution to the preprocessed signal vector, which yielded two orthogonal time series corresponding to the location of interest. Next, these virtual sensor data were decomposed into time‐frequency space and vector summed to derive a single temporal envelope of the signal corresponding to the frequency window identified through the MEG sensor‐level statistical analyses. This resulted in absolute power time series for each peak voxel per participant, which were used to examine differences in spontaneous activity during the baseline period. We then assessed whether spontaneous activity was associated with the total score from the CUDIT‐R questionnaire and reaction time during the flanker task.

### 2.12 Dynamic functional connectivity analyses

To probe dynamic functional connectivity between the cortical regions identified in the neural interference maps, phase locking was computed within the same spectral windows derived from our sensor‐level statistical analyses. Specifically, we estimated the phase locking value (PLV; Lachaux et al., 1999) between the active brain regions at the source level. The PLV reflects the intertrial variability of the phase relationship between pairs of brain regions as a function of time. Values close to one indicate strong synchronicity (i.e., phase locking) between the two brain regions within the specific time window across trials, whereas values close to zero indicate substantial phase variation between the two signals and, thus, weak synchronicity (connectivity) between the two regions. We conducted exploratory analyses to investigate the relationship between cognitive function and phase locking across brain regions supporting cognitive control. To do this, we extracted the mean PLV per participant and condition (i.e., congruent, incongruent) across the time‐frequency window used for beamforming, which we then correlated using partial correlations with the domain composite scores for the attention and executive function domains from the neuropsychological assessment and task behavior, controlling for source power, across all participants and separately by group (Arif, Spooner, et al., 2021; Schantell et al., 2024).

### 2.13 Statistical analyses

Continuous demographic and neuropsychological data were assessed using independent samples *t*‐tests, and categorical comparisons were conducted using Chi‐square tests (*χ* ^2^). Cannabis use‐by‐condition interactions and main effects of reaction time, accuracy, oscillatory gamma power extracted from peak voxels identified in regions supporting neural interference, and neural interference effects in gamma phase locking (i.e., PLV; controlling for source power) were examined using a 2 × 2 ANOVAs with a within‐subject factor of condition (i.e., congruent and incongruent) and a between‐subject factor of cannabis use status. In addition, independent samples *t*‐tests were used to evaluate group differences in spontaneous gamma activity in regions supporting neural interference. Continuous associations between oscillatory and spontaneous activity with task performance and cannabis use measures were assessed using Pearson correlations, and partial correlations were conducted to quantify the relationship between gamma phase locking and T‐scores from the executive function and attention domain composite scores from the neuropsychological assessment, controlling for source power. Further, we conducted sensitivity analyses to evaluate whether our main findings held using a 2.5 SD threshold for outlier removal. All analyses reported herein remained statistically significant using both a 2 SD and 2.5 SD threshold for outlier removal, but the results presented here reflect analyses using a 2 SD threshold. Demographic, behavioral, time series, and subsequent correlation analyses were conducted in IBM SPSS v.25.

### 2.14 Data availability policy

Requests for data can be fulfilled via the corresponding author. De‐identified data have been made available to the public through the Collaborative Informatics and Neuroimaging Suite (COINS) database.

## 3 RESULTS

### 3.1 Participant characteristics and neuropsychological results

The two groups (nonusers and cannabis users) had comparable demographic characteristics (Table 1). Groupwise comparisons of each cognitive domain revealed that cannabis users performed relatively worse on the *memory* (*p* = .039) and *executive function* (*p* = .002) domains relative to nonusers. However, cannabis users and nonusers performed similarly on the *learning*, *attention*, *processing speed*, and *motor dexterity* domains (all *p*‐values > .05).

### 3.2 Eriksen flanker task performance

To assess the relationship between cannabis use and behavioral performance during the flanker task, we conducted a 2 × 2 ANOVA. We found there was a significant main effect of condition (*F* = 93.61, *p* < .001) indicating that regardless of cannabis use status, participants responded more slowly on incongruent trials relative to congruent trials (i.e., the classic flanker effect). Neither the main effect of cannabis use (*F* = 1.16, *p* = .285) nor the cannabis use‐by‐condition interaction (*F* = 1.00, *p* = .320) were significant (Figure 1b). Regarding accuracy, there were no significant main effects in response accuracy by group (*F* = 0.91, *p* = .344), condition (*F* = 0.23, *p* = .630), or in the group‐by‐condition interaction (*F* = 0.51, *p* = .476).

### 3.3 Neural oscillatory responses

**FIGURE 2:**
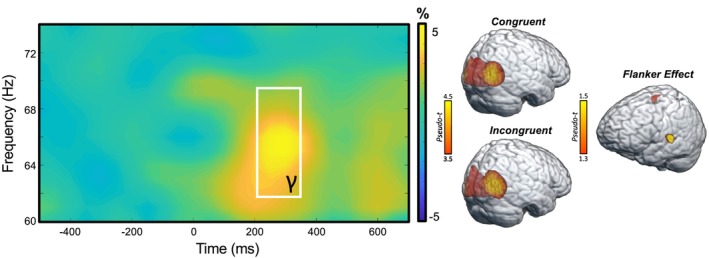
Grand‐averaged spectrogram and whole‐brain maps. (Left): Grand‐averaged time‐frequency spectrogram of an MEG sensor exhibiting a significant gamma response (62–70 Hz, 200–350 ms). The spectrogram displays frequency (Hz) on the *y*‐axis and time (ms) on the *x*‐axis. Signal power is expressed as a percent difference from the baseline period, with the color legend shown to the right of the spectrogram. (Right): Grand‐averaged beamformer images (pseudo‐*t*) across all participants for the gamma oscillatory response shown in the spectrogram for the congruent condition (Right, top), incongruent condition (Right, bottom), and flanker interference effect (i.e., incongruent—congruent; right, middle).

Sensor‐level analyses collapsed across conditions and groups revealed a significant increase in gamma power (62–70 Hz) relative to the baseline period between 200 and 350 ms (Figure 2). This window was imaged separately for each condition (i.e., congruent and incongruent) per participant. Flanker interference maps were then computed by subtracting the congruent from the incongruent map (voxel‐by‐voxel) in each participant. Participant‐level interference maps were grand‐averaged to derive a flanker interference map across all participants, which revealed clusters in the left frontal eye fields (FEF) and the left temporoparietal junction (TPJ; Figure 2, right).

**FIGURE 3:**
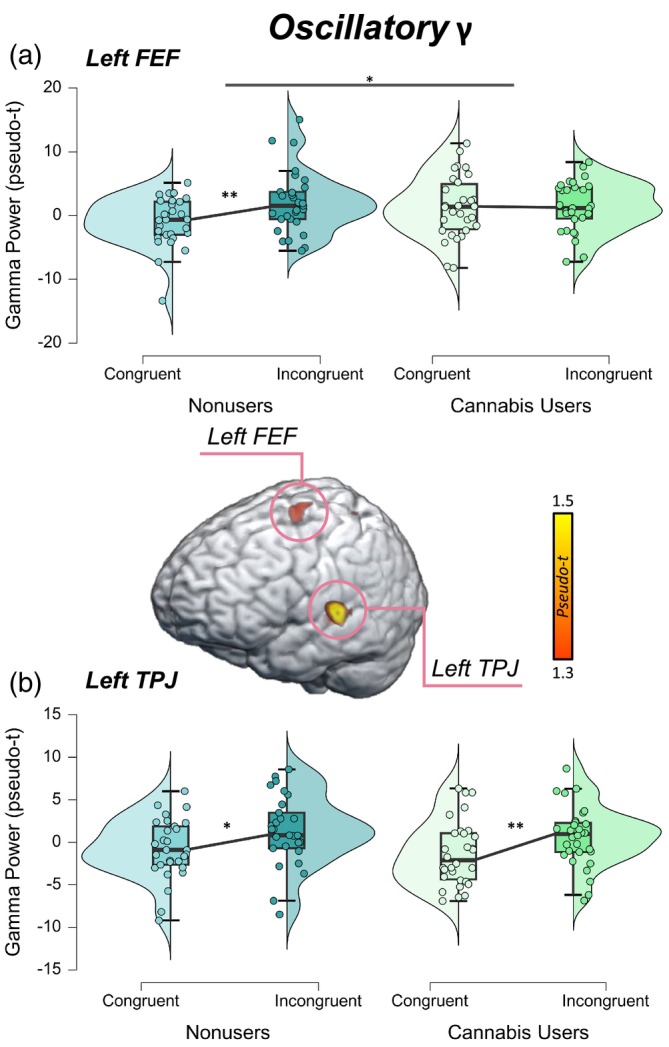
Oscillatory gamma by group and condition. Peak voxels from the whole‐brain gamma interference map were extracted separately by condition and subsequently subjected to 2 × 2 ANOVAs to assess for differences in oscillatory gamma power by condition (i.e., congruent and incongruent) and group (i.e., nonusers and users). (a) In the left frontal eye fields (FEF), there was a significant group‐by‐condition interaction in which nonusers had stronger oscillatory gamma power during the incongruent relative to the congruent condition compared to cannabis users (*p* = .042). (b) Oscillatory gamma power in the left temporoparietal junction (TPJ) was stronger during the incongruent relative to the congruent condition across cannabis users and nonusers (*p* = .002). However, there were no differences observed by group, and there was not group‐by‐condition interaction. *p < .05, **p < .01.

Next, we extracted pseudo‐t values from the peak voxels of these neural interference peaks. These data were entered into a 2 × 2 ANOVA with a within‐subjects factor of condition (i.e., congruent and incongruent) and a between‐subjects factor of group (i.e., cannabis users and nonusers). In the left FEF, there was a significant difference in oscillatory gamma power by condition (*F* = 4.32, *p* = .042) and a significant group‐by‐condition interaction (*F* = 4.32, *p* = .042; Figure 3a) in which nonusers had stronger oscillatory gamma power during the incongruent relative to the congruent condition (*t* = −2.64, *p* = .007), though this relationship was not present among cannabis users (*t* = −.001, *p* = .499). In the left TPJ, there was a significant difference in oscillatory gamma power by condition in which both cannabis users and nonusers had stronger oscillatory gamma power during the incongruent relative to the congruent condition (*F* = 10.08, *p* = .002; Figure 3b). However, neither the main effect of group (*F* = .68, *p* = .415) nor the group‐by‐condition interaction (*F* = .00, *p* = .989) were statistically significant. Of note, we removed a total of eight outliers identified above or below 2 SDs from the mean oscillatory gamma power across all participants in the left FEF and left TPJ for both conditions prior to running the ANOVAs.

### 3.4 Group differences in spontaneous activity

**FIGURE 4:**
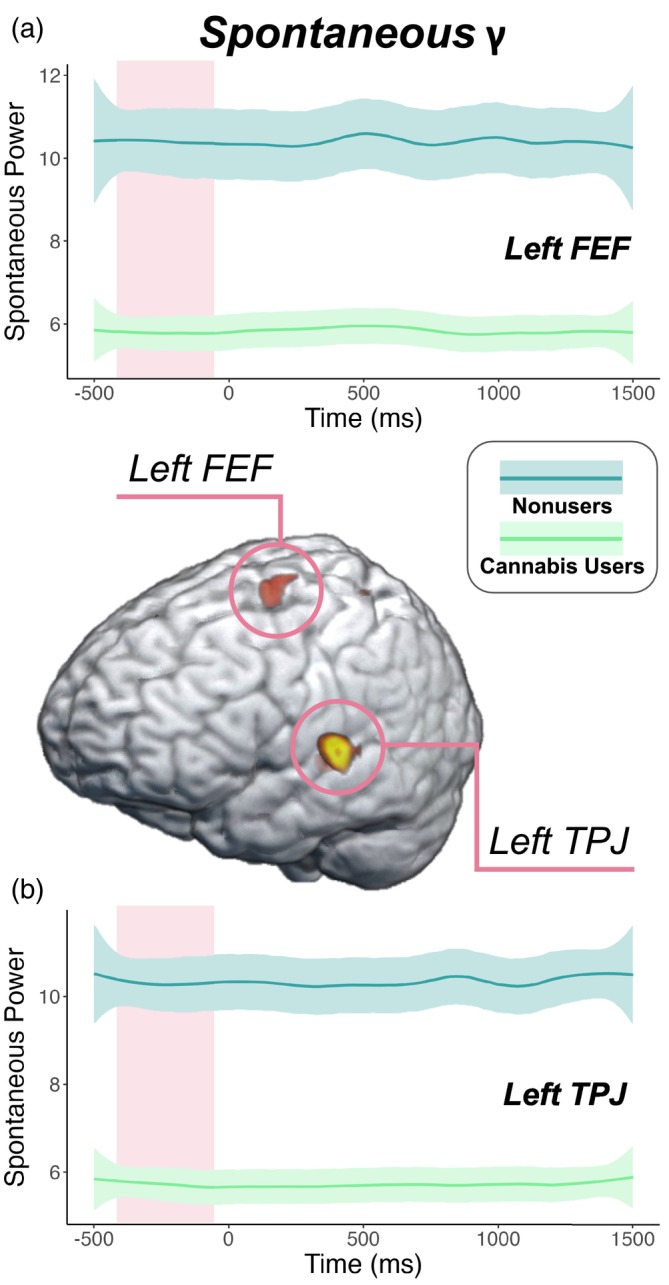
Group differences in spontaneous gamma activity. Peak voxel time series of the (a) left frontal eye fields (FEF) and (b) left temporoparietal junction (TPJ) were extracted to estimate spontaneous gamma power (nAm^2^) during the baseline period, which differed by group. Specifically, cannabis users had weaker spontaneous gamma activity than nonusers in the left FEF (*p =* .012) and left TPJ (*p* < .001). The shaded area surrounding each time series represents the standard error of the mean.

**FIGURE 5:**
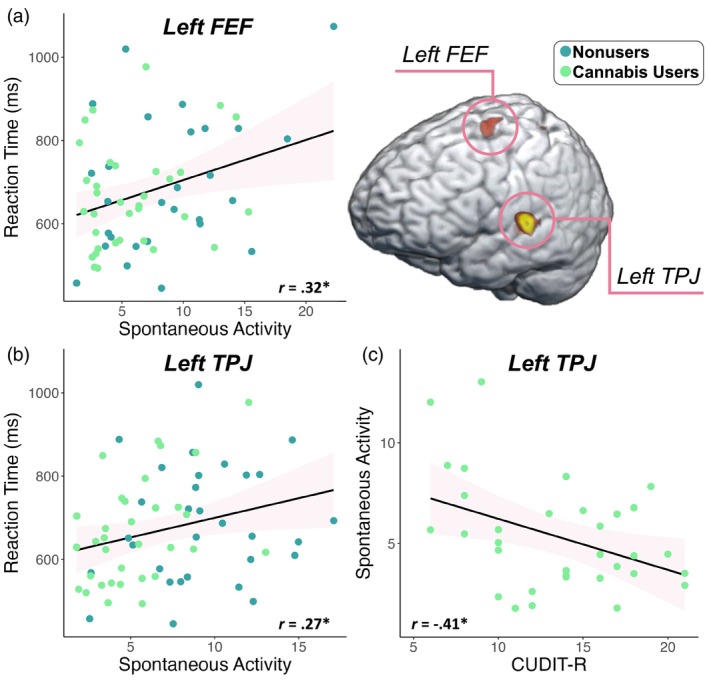
Spontaneous gamma activity scales with task performance and cannabis use metrics. (a) Weaker spontaneous gamma activity was associated with faster reaction times during the flanker task in the left frontal eye fields (FEF; *p* = .012) and (b) in the left temporoparietal junction (TPJ; *p* = .033). (c) Higher scores on the CUDIT‐R were associated with weaker spontaneous gamma activity in the left TPJ (*p* = .018). *p < .05. The pink area surrounding the trendline represents the standard error of the mean.

Next, we estimated the spontaneous gamma power during the baseline period (i.e., −450 to −50 ms) using the temporal envelope of the voxel time series data. Since these data reflect neural activity before stimulus onset in each trial, we did not expect conditional effects and thus collapsed across condition. Independent samples *t* tests of the group effect were then conducted, which revealed that cannabis users exhibited sharply reduced spontaneous gamma power in the left FEF (*t* = −2.59, *p* = .012; Figure 4a) and left TPJ (*t* = −4.92, *p* < .001, Figure 4b) relative to nonusers. Next, we assessed whether such spontaneous activity was related to behavior and cannabis use metrics using Pearson correlations. These analyses indicated that faster reaction times were associated with weaker spontaneous gamma activity in the left FEF (*r* = .32, *p* = .012, Figure 5a) and the left TPJ (*r* = .27, *p* = .033; Figure 5b) across all participants. Further, higher scores on the CUDIT‐R were associated with weaker spontaneous gamma activity in the left TPJ of cannabis users (*r* = −.41, *p* = .018; Figure 5c). Of note, there were no outliers identified above or below 2 SD from the mean spontaneous gamma power across all participants in the left TPJ, but there were two outlier data points (both nonusers) in terms of spontaneous gamma activity in the left FEF that were more than 2 SD above the mean and were removed from further analysis. Finally, to demonstrate that these differences in power during the pre‐stimulus baseline period were frequency band specific and not simply due to broadband shifts, we plotted the power spectra from 4 to 100 Hz averaged across the left FEF and TPJ by cannabis use status (Supplementary Figure 1).

### 3.5 Dynamic functional connectivity

**FIGURE 6:**
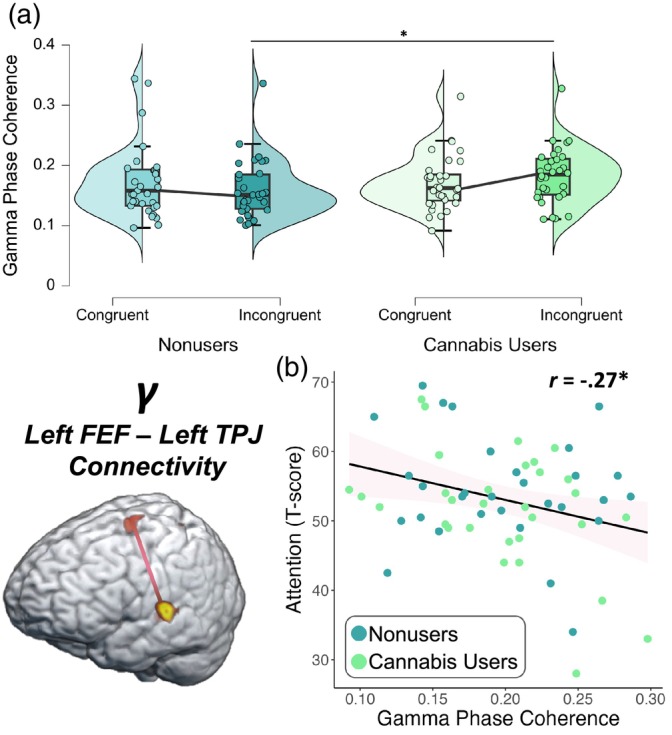
Neurobehavioral relationships in gamma phase locking between the left frontal eye fields (FEF) and temporoparietal junction (TPJ) by cannabis use status. Gamma phase locking was computed as a measure of dynamic functional connectivity between the left FEF and left TPJ. Controlling for source power, (a) cannabis users exhibited stronger gamma phase locking between the left FEF and left TPJ during the incongruent relative to the congruent condition, though this relationship was not observed among nonusers (*p* = .025). (b) Further, regardless of cannabis use status, poorer attention performance outside the scanner was associated with stronger gamma phase locking (i.e., hyperconnectivity) between the left FEF and left TPJ, controlling for source power (*p* = .037). The pink area surrounding the trendline represents the standard error of the mean. *p < .05.

Dynamic functional connectivity was evaluated using the PLV between peak voxels in the left FEF and left TPJ. Briefly, we averaged the dynamic PLV metrics during the imaging window (62–70 Hz; 200–350 ms) for each condition. These data were entered into a 2 × 2 ANOVA with a within‐subjects factor of condition (i.e., congruent and incongruent) and a between‐subjects factor of group (i.e., cannabis users and nonusers), and their interaction, controlling for source power. These analyses revealed a significant group‐by‐condition interaction (*F* = 5.29, *p* = .025; Figure 6a) whereby cannabis users exhibited stronger gamma PLV between the left FEF and left TPJ during the incongruent relative to the congruent condition, though this relationship was not observed among nonusers. Next, we correlated these values using partial correlations with each participant's executive function (i.e., phonemic verbal fluency, semantic verbal fluency, Comalli Stroop Test Interference Trial, Trail Making Test Part B) and attention (i.e., Comalli Stroop Word Reading and WAIS‐III Symbol Search) composite T‐scores derived from neuropsychological assessments outside the scanner. We found that worse attention function was associated with stronger PLV in the gamma band between the left FEF and left TPJ across both conditions and groups, controlling for source power (*r* = −.27, *p* = .037, Figure 6b). However, there was not a significant relationship between gamma PLV and the executive function domain composite score (*p* = .068). Of note, there were two outliers identified that exceeded 2 SD from the sample mean for gamma PLV and these were excluded from these analyses.

## 4 DISCUSSION

In this study, we used MEG to evaluate the relationship between chronic cannabis use and spontaneous and oscillatory gamma activity in brain regions serving cognitive control. Our main findings demonstrate that cannabis users exhibited weaker gamma oscillations in response to the interference in the left FEF relative to controls, though cannabis users and nonusers had similar oscillatory gamma interference effects in the left TPJ. Further, relative to nonusers, cannabis users had sharply suppressed spontaneous gamma activity in both the FEF and TPJ during the pre‐stimulus baseline period. We also found that weaker spontaneous gamma activity in both regions was associated with faster reaction time across all participants, and that weaker spontaneous gamma activity in the left TPJ was associated with higher CUDIT‐R scores. Of note, the CUDIT‐R is a composite measure that assesses cannabis use frequency, amount of time typically spent being under the influence of cannabis, and addiction parameters. Finally, we assessed the relationship between cognitive interference and dynamic functional connectivity between the left FEF and left TPJ among cannabis users and nonusers and found that with greater cognitive interference, only cannabis users exhibited stronger dynamic functional connectivity between the left FEF and left TPJ. To contextualize these findings, we then linked gamma phase locking with neuropsychological performance in the attention and executive function domains and found that stronger gamma phase locking overall was associated with poorer attention performance. However, performance on the executive function domain was not significantly associated with gamma phase locking between the left FEF and left TPJ. Below, we discuss the implications of these findings for understanding how chronic cannabis use modulates the neural responses serving cognitive control.

In terms of task behavior, cannabis users and nonusers performed similarly on the arrow‐based flanker task. There were no differences in task accuracy between groups, nor were there differences in accuracy by condition (i.e., congruent and incongruent). However, we did observe differences in reaction time by condition, with all participants responding faster during congruent relative to incongruent trials (i.e., the classic flanker reaction time effect). These findings are consistent with prior work using the flanker task (Embury et al., 2019; Lew et al., 2018; McDermott et al., 2017; Rangel‐Pacheco et al., 2021; Schantell et al., 2022; Taylor et al., 2021), and among other studies comparing cannabis users and nonusers (Christopher‐Hayes et al., 2021; Rangel‐Pacheco et al., 2021; Schantell et al., 2022; Springer et al., 2023). Of note, cannabis users did perform more poorly on measures of memory and executive function outside the scanner.

Our main neural findings included both oscillatory differences, as well as spontaneous differences in the time period preceding stimulus onset. Specifically, we found that cannabis users exhibited weaker oscillatory gamma interference effects in the left FEF relative to nonusers, while there were no differences in oscillatory gamma interference effects in the left TPJ by cannabis use status. Overall, these findings suggest that both cannabis users and nonusers recruited additional neural resources to overcome cognitive interference in the left TPJ, which may reflect intact neural dynamics serving bottom‐up attentional control among cannabis users and nonusers, alike. In contrast, the absence of compensatory gamma activity in the left FEF serving cognitive interference among cannabis users may reflect differences in top‐down attentional control processes related to chronic cannabis use. In addition, we found weaker spontaneous gamma activity during the pre‐stimulus baseline in the left FEF and TPJ in cannabis users, which is consistent with a previous study that found reduced spontaneous gamma activity within the somatosensory cortices of chronic cannabis users relative to nonusers (Arif, Wiesman, et al., 2021). Further, another study reported sharply reduced spontaneous gamma activity in the visual cortices of cannabis users with and without HIV relative to nonusers with and without HIV (Christopher‐Hayes et al., 2021). Similarly, other studies have found weaker spontaneous theta and alpha activity among cannabis users relative to nonusers during attention and cognitive control tasks (Schantell et al., 2022; Springer et al., 2023). Building upon this literature, we found that lower spontaneous gamma activity in the left FEF and TPJ was associated with faster task reaction times, suggesting that suppressed spontaneous gamma activity may be beneficial for cognitive control processing, at least under some circumstances. Spontaneous gamma activity in the left TPJ was also associated with cannabis use metrics in the user group. Specifically, there was a relationship between cannabis use and spontaneous gamma activity in the left TPJ, with heavier use scaling with a greater suppression in spontaneous gamma activity levels. Finally, we found that cannabis users had stronger phase locking between the left FEF and TPJ during incongruent relative to congruent trials compared to nonusers, and we found that hyperconnectivity between these regions was associated with poorer attention function across both groups.

Spontaneous and oscillatory gamma activity are of major interest given their putative role in inhibitory processing (Arif, Wiesman, et al., 2021; Cheng et al., 2016; Spooner et al., 2018; Spooner et al., 2019; Spooner et al., 2020; Wiesman et al., 2017). Essentially, gamma oscillations are widely thought to originate from the interaction of both GABAergic fast‐spiking parvalbumin inhibitory interneurons and pyramidal cells (Bartos et al., 2007; Buzsáki & Wang, 2012; Fries, 2009; Fries, 2015; Fries et al., 2007; Salkoff et al., 2015; Singer, 1999; Uhlhaas et al., 2009; Uhlhaas & Singer, 2012; Vinck et al., 2013). Animal studies have shown that CB_1_ receptors on GABAergic interneurons in the hippocampus and across the cortex are modulated by the endocannabinoid system, and thus, mediate oscillatory activity (Hajós et al., 2008; Hájos et al., 2000; Katona et al., 1999; Morgan et al., 2008; Robbe et al., 2006; Skosnik et al., 2012). Exogenous CB_1_ receptor agonists such as THC are thought to disrupt GABAergic interneurons, thereby mediating suppressed spontaneous power among acute and abstinent regular cannabis users (Böcker et al., 2010; Herning et al., 2003; Ilan et al., 2004; Skosnik et al., 2012; Skosnik et al., 2016). Further, chronic cannabis use has been related to downregulation and desensitization of CB_1_ receptors across the cortex (Bonnet & Preuss, 2017; Sim‐Selley, 2003). The PFC is particularly susceptible to these alterations given its multitude of excitatory inputs (Goldman‐Rakic, 1995; Goldman‐Rakic, 1996; Paspalas, 2005). Thus, we assessed the degree of dynamic functional gamma connectivity between the left FEF and left TPJ and how such connectivity scales with neuropsychological performance in the attention domain. We found that stronger FEF‐TPJ connectivity was associated with poorer attention performance across cannabis users and nonusers, suggesting that hyperconnectivity between these critical regions for attention may reflect neural compensatory mechanisms serving attention processing.

Before closing, it is critical to address the limitations of this study and identify future directions for this work. Using more challenging tasks that assess cognitive control in the future may illuminate more robust behavioral differences among cannabis users and nonusers, as the flanker interference task is a relatively easy cognitive task. Future work would also benefit from larger sample sizes, which would enable dose‐dependencies to be more thoroughly examined. Further, future work should investigate cannabis‐related differences in the neural power spectra and isolate whether these differences are aperiodic (i.e., reflect differences in 1/f characteristics) or periodic (i.e., putative oscillations) in nature (Donoghue et al., 2020), as such analyses may provide insight regarding the purported role that regular cannabis use plays in modulating excitatory/inhibitory balances across the cortex (Gao et al., 2017). Despite these limitations, our study revealed the relationship between chronic cannabis use and the oscillatory gamma dynamics serving cognitive control, as well as spontaneous gamma levels during the baseline, and their relationship with important cognitive and behavioral metrics.

## CONFLICT OF INTEREST STATEMENT

The authors declare that there is no conflict of interest.

## Supporting information


**DATA S1:** Supporting Information.

## Data Availability

The data that support the findings of this study are available from the corresponding author upon reasonable request.
