# Supplementary material for: Chronic cannabis use alters the spontaneous and oscillatory gamma dynamics serving cognitive control
Source: Hum Brain Mapp. 2024 Jul 18;45(11):e26787. doi: 10.1002/hbm.26787 (PMC11256138; doi:10.1002/hbm.26787)
Supplement: Supplementary file 1 — DATA S1: Supporting Information. [file HBM-45-e26787-s001.docx]

***Supplementary Materials***

*Chronic cannabis use alters the spontaneous and oscillatory*

*gamma dynamics serving cognitive control*

Mikki Schantell, Jason A. John, Anna T. Coutant, Hannah J. Okelberry, Lucy K. Horne, Ryan Glesinger, Seth D. Springer, Amirsalar Mansouri, Pamela E. May-Weeks, Tony W. Wilson


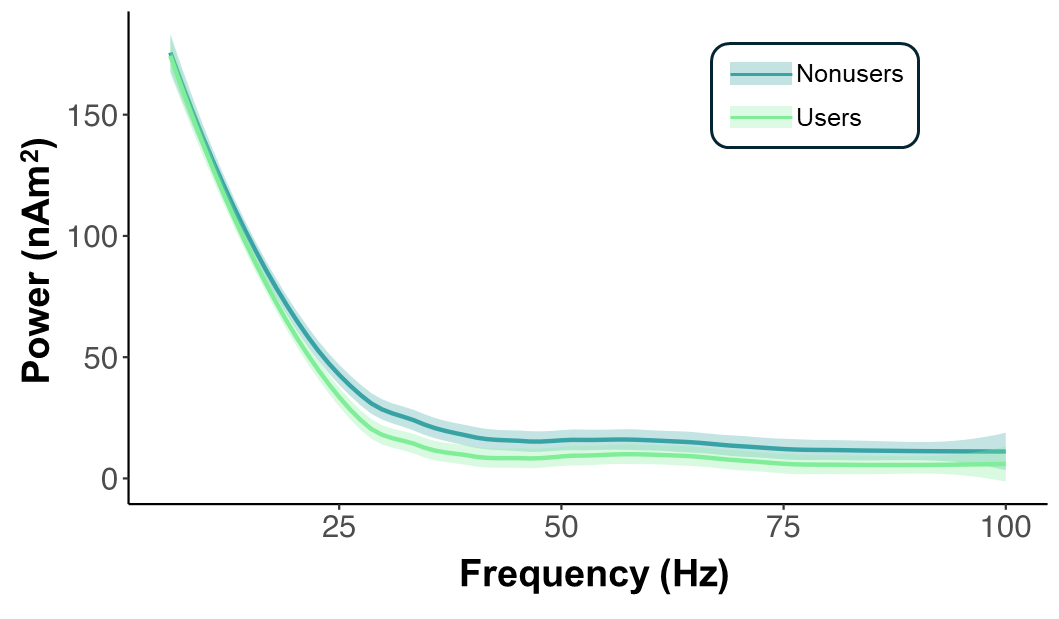


**Supplementary Figure 1. Chronic cannabis use is associated with gamma-specific differences in the power spectra across the left frontal eye fields and temporoparietal junction.** The power spectra during the pre-stimulus baseline period averaged across the left frontal eye fields (FEF) and temporoparietal junction (TPJ) shows that chronic cannabis use is associated with a greater suppression in gamma (i.e., frequencies ≥ 30 Hz) power, while power in the lower frequencies appears to be similar among cannabis users and nonusers. This suggests that cannabis-related differences in gamma power during the pre-stimulus baseline period are not simply due to broadband shifts, but instead, reflect the frequency-specific alterations in the neural dynamics that give rise to gamma activity.
